# CXCL10 and blood-brain barrier modulation in rabies virus infection

**DOI:** 10.18632/oncotarget.7428

**Published:** 2016-02-16

**Authors:** Clement W. Gnanadurai, Zhen F. Fu

**Affiliations:** Department of Pathology, College of Veterinary Medicine, University of Georgia, Athens, Georgia, USA and State-Key Laboratory of Agricultural Microbiology, College of Veterinary Medicine, Huazhong Agricultural University, Wuhan, China

**Keywords:** rabies virus, CXCL10, blood-brain barrier, Immunology and Microbiology Section, Immune response, Immunity, IL-17, tight junction proteins

Rabies is a lethal neurological disease caused by the neurotropic rabies virus (RABV). It can be prevented by prompt vaccination along with hyper-immune serum containing virus neutralizing antibodies (VNA) after a recognized exposure. However, there is no effective treatment available once the clinical symptoms appears. Various studies have shown that the laboratory-attenuated, but not the wild-type (wt), RABV can be cleared from the central nervous system (CNS), not solely due to its ability to induce innate and adaptive immunities such as production of chemokines, cytokines and VNA, and activation of immune cells, but also due to its ability to enhance the blood-brain barrier (BBB) permeability. BBB consists of a complex network of cellular system consisting of endothelial cells (ECs) which are tightly bound together by tight junction (TJ) proteins (claudins, occludin and zonula occludens-1), pericytes and astrocytes end feet, allowing selective transport of molecules to enter the CNS. Infiltration of immune effector cells from the periphery to the site of infection depends upon a cascade of events including the production of chemokines and cytokines, and modulation of BBB permeability, initiated by CNS resident cells. Thus, any abatement in the initiation and production of immune response may lead to a failure to induce BBB permeability changes and a protective immune response. Attenuated RABVs are known to induce the expression of proinflammatory chemokines and cytokines, especially those related to interferon signaling pathways, whereas wt RABVs stimulate little or no inflammatory responses [1]. Roy et al., 2007 have shown that the failure to open the BBB leads to the lethal outcome after infection with silverhaired bat RABV in mice [2]. Thus, for the effective clearance of RABV from the CNS, the presence of VNA and the enhancement of BBB permeability are required [3]. However, the mechanism by which RABV infection initiates BBB permeability enhancement was unclear.

In these issues of the *Journal of virology*, Chai et al., investigated the mechanism by which attenuated RABV infection initiates BBB permeability enhancement in mice [4, 5]. Initially, it was observed that the attenuated RABV infection in mice enhances BBB permeability by reducing the TJ proteins and inducing infiltration of inflammatory cells into the CNS. However, either attenuated or wt RABV infection did not reduce TJ proteins on brain microvascular endothelial cells (BMEC) *in vitro*, indicating that RABV infection per se is not involved in BBB modulation. It was further found that the extracts from the brains of mice infected with attenuated RABV alone, could significantly reduce the TJ proteins. Analysis of mice brain extracts showed the presence of high levels of chemokine and cytokines. Ingenuity pathway analysis of immune networks indicates that IFN-γ is the center molecule which is directly linked with CXCL10, CXCLl9, CCL5, IL-17, IL-12, IL-6 and VEGF. Likewise, the induction of innate immunity, particularly interferon mediated expression of chemokines and cytokines, and their association with BBB permeability enhancement have been reported previously [1]. Most importantly, it has been shown that the BBB permeability can be ameliorated in mice and TJ proteins can be restored in BMECs, by neutralizing IFN-γ with anti-IFN-γ antibodies, confirming the crucial role of IFN-γ on BBB permeability enhancement.

The timely initiation of chemokine network, followed by the recruitment of antigen specific T cells are crucial steps in viral clearance within the CNS. Further, it was found that that the expression of CXCL10 was highly elevated beyond the detection limit in the brain suspension of mice infected with attenuated RABV. It is found that neurons are the first to express CXCL10, as early as 3 dpi, followed by microglia and astrocytes at 6 and 9 dpi, respectively. Similarly, high level of CXCL10 induction has been observed in mice after infection with West Nile virus, Japanese encephalitis virus and Semliki forest virus [6]. CXCL10 is known to bind to its receptor, CXCR3, which is expressed in high levels in activated CD4+ T cells. Also, CXCL10 is implicated in differentiation of Th1 cells into IL-17 producing Th17 cells and IFN-γ producing Th1 cells and further governing its migration into CNS along chemokine gradient [6]. In addition to high level of CXCL10 expression, migration of significant number of IL-17 producing CD4+ T cells was found in the CNS. Further, it was demonstrated that neutralization of CXCL10 in mice diminishes the expression of both IFN-γ and IL-17, further reducing the enhancement of BBB permeability. IL-17 production has been proposed to be a key event in BBB permeability enhancement in experimental autoimmune encephalomyelitis (EAE) in mice [7]. In addition to IL-17, the BBB permeability enhancement is reinforced by amplification of CXCL10 production by IFN-γ secreted by Th1 cells through positive feedback. The detection of high level of IFN-γ expression at the late stage of infection further supports this hypothesis. Thus, these results indicate an orchestrated action of CXCL10, IFN-γ and IL-17 in attenuated RABV infection on BBB permeability enhancement. Though, IFN-γ plays a central role in reinforcement of BBB permeability enhancement, however it is clear that CXCL10 expressed in RABV-infected neurons initiates the cascade that leads to recruitment and differentiation of CD4+ T cells, reduction of TJ proteins and enhancement of BBB permeability.

**Figure 1:**
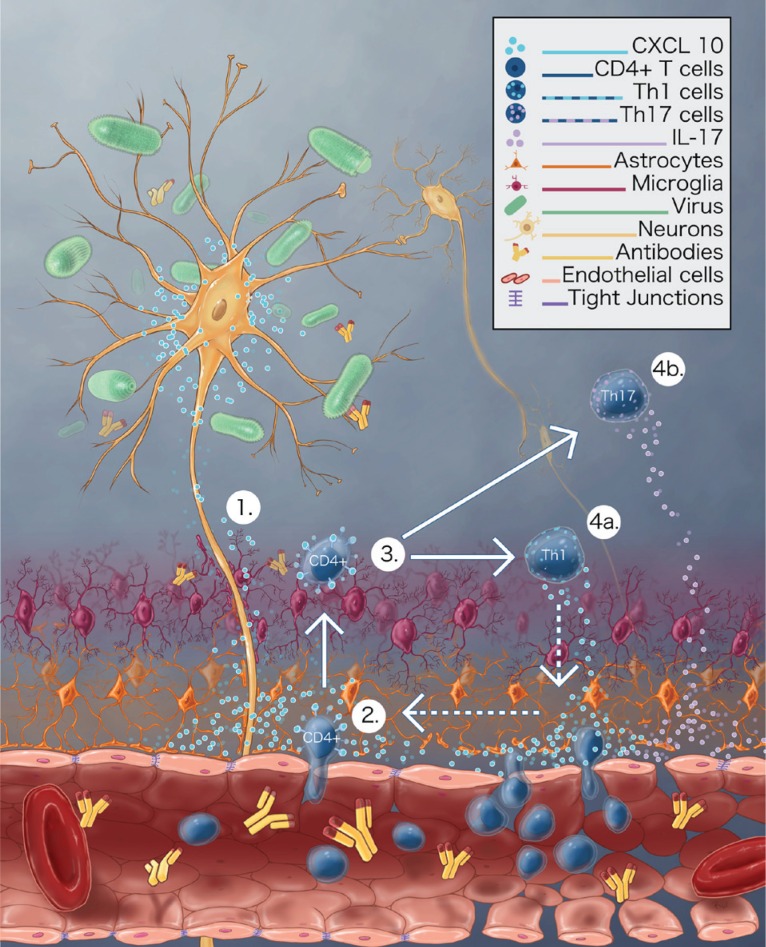
Illustration of attenuated RABV-induced neural expression of CXCL10 and BBB enhancement 1) Attenuated RABV-infected neurons secrete CXCL10. 2) CXCL10 mediates the recruitment of CD4+ T-cells into the CNS. 3) CXCL10 mediates the differentiation of CD4+ T cells into 4a) IFN-γ secreting Th1 cells, which could further boost the induction of CXCL10 through positive feedback, and 4b) IL-17 secreting Th17 cells, alters the TJ proteins resulting in BBB breakdown.

To summarize, attenuated RABV-infected neurons produce CXCL10, which leads to the recruitment of CD4+ T cells into CNS, further differentiating into Th1 cells and Th17 cells. IFN-γ producing Th1 cells boost the induction of CXCL10 through positive feedback, whereas the secreted IL-17 alters the TJ proteins resulting in BBB breakdown (Figure 1). BBB permeability enhancement is one of the crucial steps associated with RABV clearance from the CNS, which allows the passage of immune effectors from the periphery into the CNS. Thus, understanding the mechanism of BBB permeability enhancement would pave way for the development of effective therapy for clinical rabies.
